# Evaluating the Haemodynamic Performance of Endografts for Complex Aortic Arch Repair

**DOI:** 10.3390/bioengineering9100573

**Published:** 2022-10-18

**Authors:** Sampad Sengupta, Yu Zhu, Mohamad Hamady, Xiao Yun Xu

**Affiliations:** 1 Department of Chemical Engineering, Imperial College London, South Kensington Campus, London SW7 2AZ, UK; 2 Department of Surgery & Cancer, Imperial College London, St. Mary’s Campus, London W2 1NY, UK

**Keywords:** thoracic endovascular aortic repair, haemodynamic, branched stent-graft, computational fluid dynamics

## Abstract

Thoracic endovascular aortic repair (TEVAR) of aortic aneurysms and dissections involving the arch has evolved over the last two decades. Compared to conventional surgical methods, endovascular repair offers a less invasive treatment option with lower risk and faster recovery. Endografts used in TEVAR vary in design depending on the procedure and application. Novel endografts (e.g., branched stent-graft) were developed to ensure perfusion of blood to the supra-aortic vessels, but their haemodynamic performance and long-term durability have not been adequately studied. This review focuses on the use of computational modelling to study haemodynamics in commercially available endografts designed for complex aortic arch repair. First, we summarise the currently adopted workflow for computational fluid dynamics (CFD) modelling, including geometry reconstruction, boundary conditions, flow models, and haemodynamic metrics of interest. This is followed by a review of recently (2010-present) published CFD studies on complex aortic arch repair, using both idealized and patient-specific models. Finally, we introduce some of the promising techniques that can be potentially applied to predict post-operative outcomes.

## 1. Introduction

Aortic arch surgery has come a long way from its first reported case in 1964, with modern methods often combining conventional measures and novel hybrid procedures. Surgeons are continually aiming to develop new and less-invasive methods to carry out aortic arch treatment, which are now possible due to technological advancements in endograft design and deployment methods [1,2]. However, certain haemodynamic parameters of interest cannot be measured in vivo, necessitating the use of computational modelling to provide greater insight into endograft-induced haemodynamic changes. Flow related parameters such as velocity, pressure, wall shear stress, and helicity are of primary interest in determining the haemodynamic performance of endografts. Whilst blood velocities can be obtained by using phase-contrast magnetic resonance imaging (PC-MRI) or 4D flow MRI [3,4], it is difficult to extract velocity gradient-based metrics directly and image-based computational modelling has been shown to provide reliable predictions of haemodynamics in diseased and post-TEVAR aorta [5,6,7]. This review aims to dive into the use of computational methods to simulate and investigate the performance of endografts used for aortic arch repair, whilst also touching upon the performance of some existing devices.

### 1.1. Aortic Arch Disease

**Figure 1:**
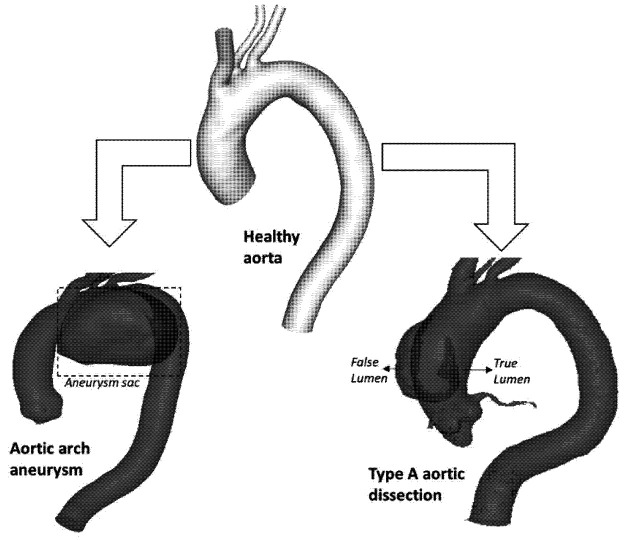
Schematic illustration of common aortic diseases in the ascending aorta and aortic arch.

Common diseases of the aortic arch include aneurysms and dissections, as shown schematically in Figure 1. An aneurysm manifests as permanent dilatation of a portion of the arterial wall. The formation of an aneurysm is multifactorial and primarily degenerative, resulting in gradual weakening and thinning of the vessel wall. Expansion of the aneurysm may eventually cause it to rupture, which can be fatal [8]. The British Heart Foundation estimates that approximately 5000 deaths are caused by ruptured aortic aneurysms every year in the UK. Aneurysms in the aortic arch are rare when compared to abdominal aortic aneurysm (AAA), with an incidence of around 5–10 cases per 100,000 patients per year [9]. The development of an aneurysm increases the risk of aortic dissection, which is another life-threatening aortic disease, with a higher occurrence rate than that of ruptured AAA in the western world [10]. An aortic dissection is characterised by a tear in the vessel wall which causes the formation of a new conduit referred to as the false lumen (FL) alongside the true lumen (TL). Acute aortic dissections can lead to aortic rupture within a matter of minutes or hours, after which the patient’s risk of death increases 1% per hour and thus calls for urgent open surgery [11].

The conventional procedure for the treatment of aortic arch pathologies is open surgery, which involves replacing part of the affected aorta with a synthetic graft. During the procedure, the diseased aorta segment is removed and replaced through open-chest surgery, a highly complex operation with high blood loss, huge fluid shifts and a massive surgical trauma. Modern surgery is always accompanied by extracorporeal circulation (cardiopulmonary bypass or heart-lung machine) to reduce the risk of cerebral ischemia. Despite advances in surgical techniques, a significant mortality rate of 7–17% has been reported, while the occurrence rate of neurological injury ranges from 4–12% [12]. The complicated nature of the procedure when dealing with the aortic arch gives rise to more modern and less invasive treatment approaches such as endovascular aortic repair (EVAR).

### 1.2. Thoracic Endovascular Aortic Repair

Thoracic endovascular aortic repair (TEVAR) offers a less invasive approach when compared with open surgery since there is no need for large incisions and long periods of interrupting blood flow. It can also be combined with previous open and hybrid techniques to minimise the risks involved and potentially improve patient outcomes. The development of new branched endografts used in aortic repair has now somewhat quelled the debate between open and hybrid repair techniques [13,14,15,16]. Recent studies have demonstrated distinct advantages of TEVAR over open surgical repair, including less peri-operative and short-term mortality, less time in intensive care and hospital, and a quicker recovery [17]. Examples of some arch and thoracic endografts are the Valiant Mona LSA (Medtronic Vascular, Santa Rosa, CA, USA), cTAG and Gore TBE (W.L. Gore & Associates, Flagstaff, AZ, USA), Zenith (William Cook, Bjaeverskov, Denmark), and Nexus^TM^ (Endospan, Herzlia, Israel). Nowadays, TEVAR has become one of the most popular choices for treating descending thoracic aortic disease and in the regions of the aortic arch [12,18,19].

#### 1.2.1. Challenges for TEVAR

Endovascular repair using endografts (or stent-grafts) always requires a sufficiently long length of healthy aorta of at least 2 cm as a proper landing zone to achieve adequate fixation of the device to prevent migration. However, this proves to be difficult in the aortic arch due to its angulated morphology and the presence of three supra-aortic vessels, which originate fairly close to each other. Intentional covering of some of the arch vessels can extend the proximal landing zone but may cause other adverse consequences, such as stroke, upper extremity and cerebral ischemia [20,21]. Therefore, the major challenge is to preserve blood flow to the supra-aortic vessels while ensuring a sealing zone for the device [22]. Moreover, as the proximal landing zone approaches an increasingly curved portion of the arch, the risk of misalignment increases as the relatively rigid stent-graft finds it harder to follow the inner curvature of the aortic arch and results in bird beak configuration [23]. To supply sufficient blood perfusion to brain, several recently developed techniques include (i) hybrid repair by combining the debranching procedure with endografting, and (ii) branched or fenestrated stent-grafts. Some of these have been discussed in the latter sections of this review.

**Figure 2:**
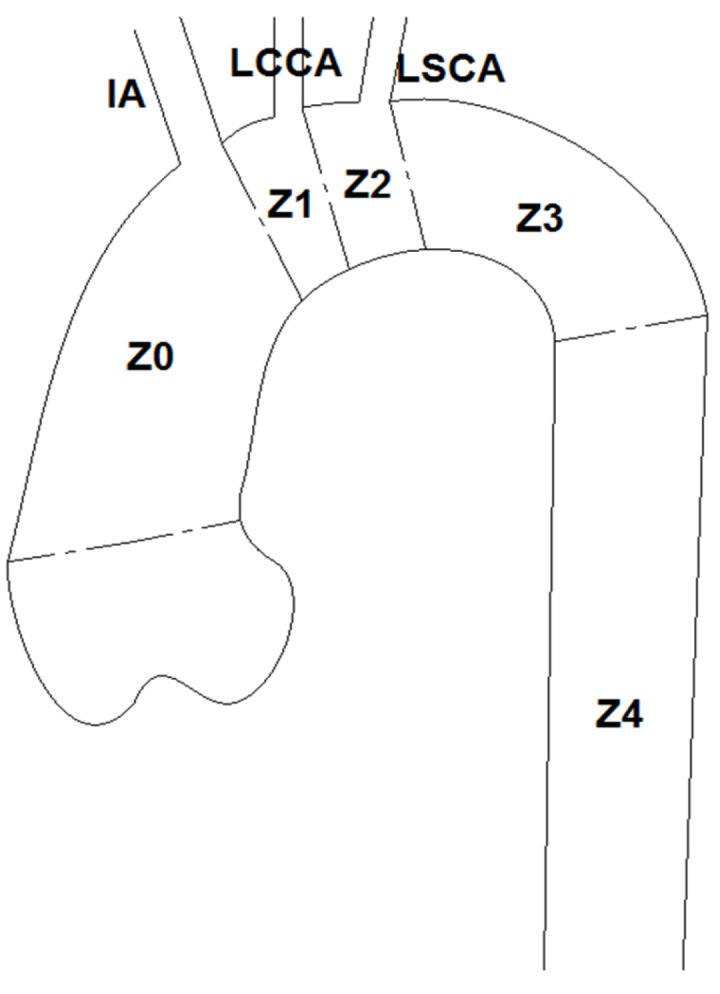
Anatomical map of landing zones in the aortic arch. Note that IA is also known as brachiocephalic artery (BCA) in some literature.

Endografting of the aortic arch can be classified based on the locations of the proximal landing site, as described by Balm et al. [24]: Z3 if in the distal arch, Z2 if distal to the Left Common Carotid Artery (LCCA) region while the Left Subclavian Artery (LSCA) is occluded by the device, Z1 is located immediately distal to the Innominate Artery (IA), also commonly referred to as the brachiocephalic artery. This means both the LCCA and LSCA are occluded and Z0 is located in the ascending aorta, thus blocking all of the three supra-aortic vessels (Figure 2).

Hybrid aortic arch repair combines the use of TEVAR with revascularisation of the supra-aortic vessels, usually incorporating a LSCA bypass through the LCCA. Although this method requires open surgery in the neck to perform the bypass procedure for revascularisation of the upper vessels, it is less invasive than that of open chest surgery with no need for circulatory arrest time [8,25,26]. Several groups compared open surgery with hybrid repair and found that there was no difference in peri-operative or 30-day mortality rates between these two procedures, but the latter carried a higher rate of re- intervention (1% vs. 20%) within three years. Based on these studies, they concluded that hybrid aortic arch repair should be performed mainly on high-risk patients who are unsuitable for open surgical repair [27,28,29].

#### 1.2.2. Endografts

Endografts used for aortic arch repair are designed to mimic a patient’s anatomy as closely as possible. They can be branched or unbranched, generally dependent on the zone of the aorta in which they are deployed. Several manufacturers have developed different varieties of endografts depending on the requirements of clinicians and their applications. Developments have been made to the design and deployment methods of endografts yielding a remarkable decrease in mortality and morbidity rates of the repair procedure [30].

As mentioned previously, branched or fenestrated stent-grafts are often used to ensure perfusion of blood to the supra-aortic vessels. Fenestrated stent-graft (FSG) is a customized device where each fenestration on the main endograft corresponds to an ostium of the supra-aortic vessels. Although FSGs work well in clinical applications, they involve a time delay for preparation and therefore these devices are not only expensive but also unsuitable for urgent cases. Branched stent-grafts are conceptually more appealing than FSG as they are adaptable to a wide range of anatomical morphologies. Branched stent-grafts can be manufactured as either single- or multi- branched endografts with or without inner tunnels. These inner tunnels can be either antegrade or retrograde. Single-branched stent-graft requires two bypass connections between the upper branches, e.g., bypass between the innominate artery and left common carotid artery or between the left subclavian artery and the left common carotid, and thus may result in insufficient blood perfusion to the supra-aortic arteries as the entire flow is supplied by a single bridging stent. Double-branched endografts are developed for Z0 deployment with two bridging stents connected to the innominate and left common carotid arteries [31,32,33].

The current generation of endografts often encounter problems that are common to a number of similar devices. Deliverability and fixation prove to be problems arising in the surgical procedure. The aim to be minimally invasive has led to complications in the delivery process which have caused ruptures in the vessel wall or inaccurate deployment. This then leads on to the issue of fixing these endografts in place. Early endografts depended on the friction due to radial force and strength of stents to hold the grafts in place, However, a number of modern devices have improved fixation methods to anchor them in place, thereby decreasing the cases of endograft migration in the vessels [30,34,35].

A persistent problem with endografts continues to be the occurrence of endoleaks. This is when blood leaks into a gap between the endograft and vessel wall, commonly the aneurysmal sac. This can lead to expansion of the aneurysm or even its rupture and requires repeat surgery. The proper fixation of an endograft, consequently held in place, and aligned with the vessel wall would reduce the incidence of endoleaks. This leads us on to the flexibility or lack thereof of the device, in relation to the aortic wall. The vessel wall is compliant and undergoes periodic changes throughout the cardiac cycle. A rigid endograft results in poor alignment or sealing of the vessel region in question, resulting in endoleaks, migration and potentially stent erosion. Several devices, in addition to material considerations, often utilise proximal and distal stents to allow for better fixation and thereby accommodating for changes in the shape of the aorta throughout the cycle. These are designed to improve the durability of the graft, thereby minimising the need for follow-ups and monitoring [35,36].

**Figure 3:**
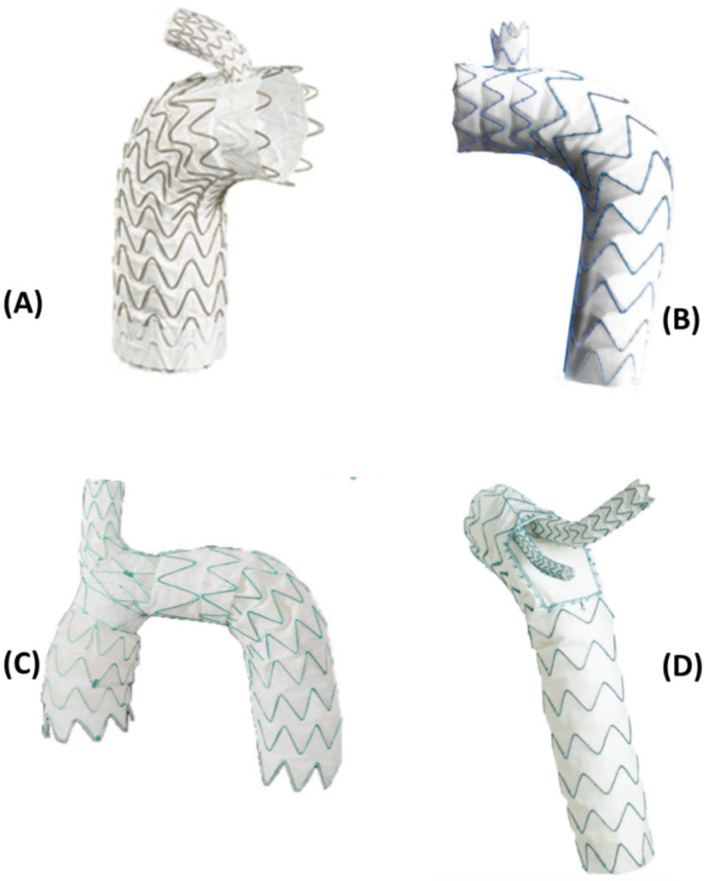
Endografts included in this review, covering a range of deployment zones and configurations. (**A**) Gore TAG endograft, (**B**) Castor Aortic Branched Stent-Graft (both single-branched, Zone 2 deployment), (**C**) Nexus^TM^ endograft (Single-branched, Zone 0 deployment), (**D**) RelayPlus endograft (Double-branched with inner tunnel branches, Zone 0 deployment) [2,34,38].

In this review, we take a look at some commercially available endografts (Figure 3) and briefly summarize their design and performance (Table 1).

**Table 1:** A brief overview of some commercially available endografts.

Device	Manufacturer	Stent/Graft Material	No. of Branches	Landing Zone	Description
Inoue Stent Graft [37]	PTMC institute	Nickel titanium/Dacron	1–3	0–2	The ISG consists of a main body with up to three branches attached separately based on axial location of target vessels. The landing zone is dictated by the number of emerging branches and vice versa in order for cuffed rings to be able to secure the graft in place both proximally and distally.
RelayPlus [2,30,38]	Terumo Aortic	Nitinol/Polyester	2	0	The RelayPlus double-branched device is formed of three main components: the main graft body and the two branches extending out via a covered window in the superior aspect of the main body (incorporating the Relay^®®^Branch system). Tunnel branches originating in the proximal end and running along the device wall connects to the emerging branches which lead into the IA and LCCA.
Zenith Arch Branched Graft [30,39]	Cook medical	Nitinol/Polyester	2	0	The Zenith arch branched graft, as the name suggests is a branched endograft that can be used for Z0 endovascular repair. It consists of two inner branches that serve to preserve blood flow to the supra-aortic vessels. It is custom-made specifically for arch repair, with reportedly acceptable morbidity and mortality rates.
Nexus^TM^ Stent-Graft [30,34,39]	Endospan	Nitinol/PTFE	1	0	The NEXUS^TM^ Aortic Arch Stent Graft System is a single-branched endograft allowing for Z0 fixation in the proximal aortic arch. It consists of a main module for the aortic arch and descending aorta with a side-branch for one supra-aortic vessel and a curved module for the ascending aorta that connects to the main module through a self-protecting sleeve and lands into the sinotubular junction.
Gore TAG/TBE [2,30]	W L Gore & Associates	Nitinol/PTFE	1	2	The Gore TAG device is a single-branched endograft designed primarily for Z2 deployment. It then served as inspiration for the Gore TBE (Thoracic Branch Endoprosthesis), aimed at treating distal aortic arch aneurysms. It consists of a main graft with a side-branch component. The side-branch is tapered with a retrograde orientation extending into the main graft body.
Valiant Mona LSA [30,34]	Medtronic	Nitinol/Polyester	1	2	The Valiant Mona LSA is a single-branched aortic endograft primarily for Z2 deployment. The functional sizing of the graft avoids the need for fenestrations as well as not covering the LSCA. The device consists of two components: a main stent graft (MSG) and a branch stent graft (BSG). This allows maintenance of LSCA perfusion and are arranged such that it has minimal thickness, thereby providing maximum lumen area.
Castor Aortic Branched Stent-Graft [2,30,34]	MicroPort Medical Co., Ltd.	Nitinol/Polyester	1	2	The Castor Aortic Branched Stent-Graft is a unibody endograft which an emerging branch that allows for LSCA vascularisation. It allows for Type B Aortic Dissection treatment with a Z2 by excluding the proximal entry tear whilst perfusing the LSCA.

The design and configuration of the endografts are often dependent on their application. As can be seen in Figure 3 there exists different kinds of branched endografts used in the arch. Not only must considerations be made to the design of the device, but also with regard to the choice of materials for different components of an endograft. The most common material for the metallic stent component is nitinol, while cobalt-chrome and stainless steel have also been used. Super-elasticity (or pseudoelasticity) of the material in response to an applied stress is an essential property in manufacturing stents. This coupled with the shape memory effect that allows alloys to return to their original shape makes these materials highly desirable for manufacturers to use in the stent [30,40]. The graft component is commonly made of polyester, Polytetrafluoroethylene (PTFE), ePTFE (expanded PTFE) or Dacron. Biocompatibility of the graft material is paramount in case of selecting a material for the graft. The host tissue response to the material used is vital in order to ensure there is no undesirable inflammatory reaction once the endograft is deployed. PTFE and Dacron exhibit lower thrombogenesis than polyester, providing an ideal material for the graft body to restore regular flow in the lumen [40].

## 2. Modelling Methodology

Here, we give an overview of the methodology commonly used for computational haemodynamics modelling and an insight into the pre-processing stages of carrying out patient-specific simulations. The parameters of interest in determining the haemodynamic response of endografts are also briefly outlined.

### 2.1. Image Acquisition and Geometry Reconstruction

All clinical diagnoses and treatment are closely aligned with medical imaging; this imaging data is crucial to developing anatomically accurate 3D geometries to serve as the computational domain. Common imaging modalities include Computed Tomography (CT), MRI, ultrasound, and X-ray angiography. Each of the above have their own merits but the primary necessity in the case of computational modelling is to ensure sufficient anatomical details to allow for accurate segmentation and thereby, reconstruction of the geometry. Non-invasive imaging methods such as echocardiography, phase-contrast MRI and 4D Flow MRI can provide flow-related measurement data which can be used to define patient-specific simulation conditions.

The raw DICOM data acquired from imaging often contains very large sets of multislice data. This can be analysed using 3D image processing software, which involves a series of automatic and manual segmentation steps to generate a representation of the region of interest. The relevant anatomical features from CT data, for example, are isolated from the imaging field by thresholding techniques to filter out information beyond the bounds of a set grayscale intensity. Once initial 2D masks are generated through all the slices of the original dataset, a region growing tool is applied, allowing recruitment of the relevant neighbouring pixels. An iterative process of refining the obtained 2D masks is necessary before creating a 3D luminal surface. This is then used to set up accurate 3D in silico geometries which provide the physical boundaries for the model. It can be a time-consuming process, especially when dealing with a large cohort of patients. Thus, attempts are being made to automate the image segmentation and 3D reconstruction process for improved accuracy and efficiency.

### 2.2. Computational Methods

**Figure 4:**
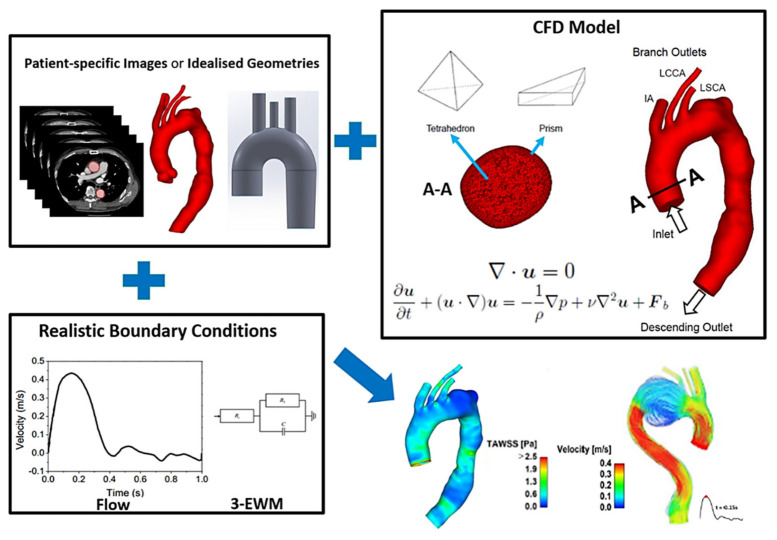
Schematic of the key stages involved in computational flow modelling of the aortic arch.

Figure 4 gives an overview of the workflow involved in performing computational haemodynamics simulations. The fluid volume of interest is defined by the 3D geometric model, either created based on idealised dimensions and conditions or obtained from clinical images as mentioned previously. Blood flow through the reconstructed geometric model is governed by the conservation of mass and momentum equations which are partial differential equations. Solving these equations requires the use of computational fluid dynamics (CFD) techniques. Briefly, the fluid domain is discretised into a large number of small computational cells, a step known as mesh generation which can be performed using a wide range of available software. The relevant conservation equations are then discretised and solved on the generated computational mesh. Mesh refinement is a key step when setting up a CFD model as the quality and density of a mesh dictate the accuracy and stability of the simulation results, whilst also affecting the computation time required to achieve a converged solution. Coarse meshes with large grid sizes will produce inaccurate results and thus it is essential to refine the mesh until the numerical solution becomes mesh independent. Combined with suitable boundary conditions, the partial differential equations are solved and approximate solutions for velocities and pressures are obtained. These simulations can be performed using a variety of open source or commercially available CFD software, each presenting with its own advantages and disadvantages and is often chosen based on the application or the preference of the user.

The preferred inlet boundary condition is a patient-specific velocity profile obtained from phase-contrast or 4D flow MRI or Doppler ultrasound, but this data is not always available. Therefore, generic boundary conditions such as representative flow waveforms along with the assumption of flat or fully developed velocity profiles are commonly used. Typical aortic flow waveforms can be found in the literature [41]. When it comes to outlet boundary conditions, pressure information is required to numerically solve the mass and momentum conservation equations and for the CFD solver to be robust and stable. The most common outflow boundary conditions include constant pressure, prescribed flow split, a lumped constant resistant model and Windkessel models. The fixed flow-split and constant pressure options are easy to implement but fall short in terms of providing physiologically accurate solutions, especially in pressure-dependent results. This drawback can be overcome by using the Windkessel model instead. The 3-element Windkessel (3-EWM) model is considered a reliable outlet boundary condition as it can capture the characteristics of the downstream vasculature and thus provide a relatively accurate prediction of pressure results [42]. Since a rigid wall assumption is usually made in majority of the computational flow studies, a no-slip condition with zero velocity is specified at the walls.

Blood is a complex fluid and is a combination of red blood cells, white blood cells, platelets and other agents suspended in a fluid plasma. It therefore tends to exhibit non-Newtonian characteristics with a shear-dependent viscous behaviour. Blood is a shear-thinning fluid, which means that the viscosity of blood decreases as the shear rate increases. Some of the most commonly used non-Newtonian blood flow models include power law model [43], Casson model [43], Quemada model [44], and Carreau model [43,44,45]. On the other hand, it has been widely accepted that blood can be assumed as a Newtonian fluid in large vessels where shear rate is usually greater than 100 s^−1^ [46,47]. Previous numerical studies have shown that the Newtonian assumption was adequate for arterial flow since differences in the results of Newtonian and non-Newtonian simulations were insignificant [48,49]. However, as there are regions of flow stagnation in aortic aneurysm where local shear rate can be less than 10 s^−1^, the non-Newtonian effect of blood could be important in localized regions. Whilst a Newtonian assumption is often sufficient for large vessels, non-Newtonian models have been adopted in cases where the simulated process (e.g., thrombus formation) is sensitive to changes in blood viscosity [50]. Hence, if information on patient-specific haematocrit is available, the non-Newtonian viscous behaviour of blood should be included by using one of the established non-Newtonian models.

Flow can be characterized as laminar, transitional or turbulent based on the Reynolds number (*Re*) of the flow. Re is defined as the ratio of inertial forces to viscous forces which can be expressed as $\;Re=\frac{\rho UD}{\mu }$, where *U* is the mean velocity, μ is the dynamic viscosity which is 0.004 Pa.s for blood and *D* is the vessel diameter [51]. For steady flow in a straight, circular pipe, the critical Re for transition to occur was found to be 2000, below which the flow could be considered laminar. Once Re exceeds 2000, the inertial forces in the flow become sufficiently large compared to the viscous forces, breaking down laminar flow first to a transitional state and then turbulent. In real arteries, however, the critical Re for blood flow to transition can also be influenced by other factors, such as arterial wall roughness, upstream disturbances, and spatial and temporal retardation [52]. In the human body, blood flow in large vessels is usually assumed to be laminar since the mean velocity is low enough to yield relatively low *Re*. However, blood flow may be transitional in the presence of heart valve pathologies or in diseased arteries, in cases such as coarctation of the aorta and severe carotid stenosis [53]. An experimental study on canine aortas was conducted by Nerem et al., who found that the presence of disturbed blood flow can be decided by the peak Reynolds number (Re^) and Womersley number (α). Stability boundaries were derived for the ascending (Re^ = 150α) and descending (Re^ = 250α) aorta using these parameters [54].

### 2.3. Haemodynamic Metrics

The physical quantities calculated via computational simulations can be used to generate a range of indices that cannot be measure in vivo. The metrics utilised in each study is dictated by its need and application and a list of commonly used haemodynamic metrics is given in Table 2.

Large displacement forces acting on the stent-graft are the main cause for distal migration of the device [55], which may further lead to type I endoleak, thereby increasing the risk of aneurysm rupture. Thrombus formation within the stent-graft can result from high PLAP, OSI, RRT, and ECAP magnitudes [40,55,57]. Low WSS is also known to promote thrombus formation by facilitaing platelet adhesion [62], whereas high WSS values are associated with platelet activation [63], arterial wall thinning and even rupture [64,65]. Flow patterns can be described by either HFI, vorticity, or turbulence intensity, with higher values representing greater level of flow disturbance.

## 3. CFD Analysis of Aortic Arch Repair

In this section we review some of the CFD studies carried out for aortic arch repair, touching upon both idealised and patient-specific models. This aims to give an overview of the methods employed in aortic arch modelling and the scope of its abilities. Studies included here are based on a selection criterion in order to ensure it remained pertinent to the focus of this review. Pathologies included here were aortic arch aneurysms and ascending aorta and arch dissections, treated with TEVAR with landing zones within Z0 to Z2. The timeframe was restricted to studies between 2010-present, which allowed for maintaining relevance and the use of modern computational methods.

### 3.1. Idealised Models

A few CFD studies have been reported for evaluation of the haemodynamic performance of endografts for aortic arch repair using idealised models [15,36,66,67]. Nardi and Avrahami compared the haemodynamic performance of different treatment approaches for an aortic arch aneurysm, including surgical repair, hybrid approach and chimney technique. Their study showed that surgical repair exhibited the best haemodynamic performance, while the chimney procedure produced the maximum WSS and pressure drop, together with highly disturbed and vertical flow [15]. The haemodynamic performance of a novel stent-graft design with slit perforations were assessed [36,66]. By comparing two endograft configurations with full- and half-slit design, Ong et al. found the effect of slit design to be minor [36]. Another study carried out by Liu et al. investigated the haemodynamic performance of a single-branched endograft with 4 different LSCA branch stent configurations. Among the others, the design with a curved cuff branch performed the best with lower regions being exposed to low WSS, and high RRT and OSI. The protruding branch configuration considerably reduced blood supply to the LSCA and should therefore be avoided [67].

**Figure 5:**
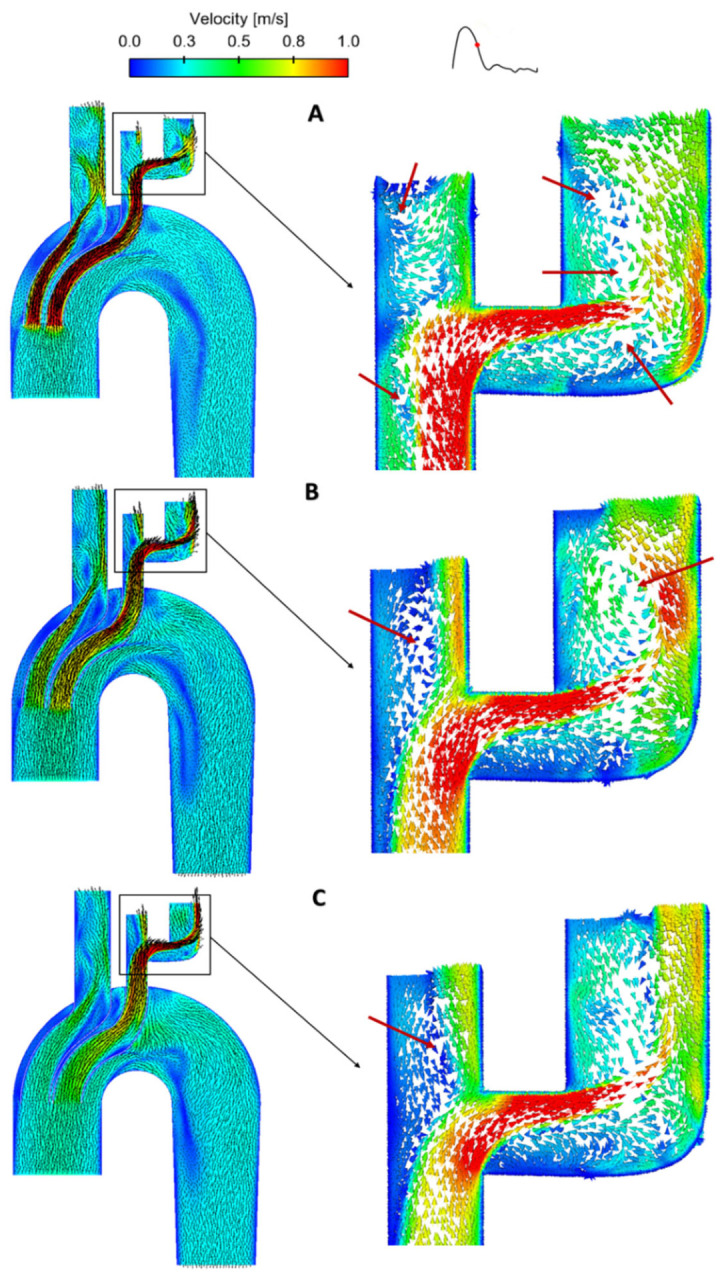
Comparison of velocity contours in the sagittal plane along with in-plane velocity vectors between the three BSG models: (**A**) Stent-graft 1, (**B**) Stent-graft 2, and (**C**) Stent-graft 3, at mid-systolic deceleration (0.29 s). Detailed flow patterns are shown by local enlargement of the LCCA-LSCA bypass (right column). FRZs in the bypass graft are indicated by red arrows.

In what follows we present results obtained from our previous simulation work on idealised models for a double-branched stent-graft with two equal-diameter inner tunnels within the main graft body [68]. The idealised aortic arch model was created based on the physiological dimensions reported by Finlay et al. (2012) [69]. It was planar with representative diameters for the supra-aortic branches (15 mm for IA, 9.5 mm for ICCA and 13 mm for LSCA). This simplified model was employed to investigate the effects of varied inner tunnel diameters on the haemodynamic performance of the device. For this purpose, three hypothetical models were built with inner tunnels’ diameters being 8 mm, 10 mm and 12 mm, respectively, and the corresponding models (shown in Figure 5) were named as stent-graft 1, stent-graft 2, and stent-graft 3. It’s worth noting that the tunnel branch in stent-graft 2 matches closely the diameter of LCCA, whereas the tunnel branch in stent-graft 3 better matches the IA diameter. In addition, the LCCA-LSCA bypass was included in the hypothetical branched stent-graft models.

To assess the effectiveness of branched stent-graft for TEVAR, it is essential to understand how it may affect blood flow to the arch vessels. Therefore, the cycle-averaged volumetric flow rates at the four outlets of all the simulated models were determined, together with the corresponding percentages of flow split. In the aortic arch model without a stent-graft, flow distribution to the IA, LCCA and LSCA was 13.6%, 5.4% and 10.4%, respectively. The implantation of a double-branched stent-graft slightly reduced the amount of flow through the IA (by 0.2–0.3%), slightly increased flow to the LCCA (by up to 0.3%) and LSCA (by up to 0.9%) except for stent-graft 1 which has the smallest inner tunnel diameter.

For detailed evaluation of flow, a cross-sectional view of the stent-graft model in the sagittal plane was plotted with velocity contours along with in-plane velocity vectors as shown in Figure 5. Blood flow accelerated when entering the inner tunnels due to the reduced cross-sectional area, which was more prominent in stent-graft 1. Moreover, there were gaps in between the two inner tunnels as well as between the tunnels and the endograft body, where blood velocities appeared to be quite low. Looking at the arch branches, flow recirculation zones (FRZ) could be observed in the LCCA and the bypass graft, as highlighted by red arrows. FRZs correspond to low WSS, which could increase the possibility of thrombus formation and should be minimised or avoided whenever possible [60,68]. Changing the inner tunnel diameters had a strong influence on local flow patterns inside the arch vessels, where the size and strength of FRZs varied. Stent-graft 3 with the largest inner tunnel diameter performed better with only one FRZ located in the LCCA.

**Figure 6:**
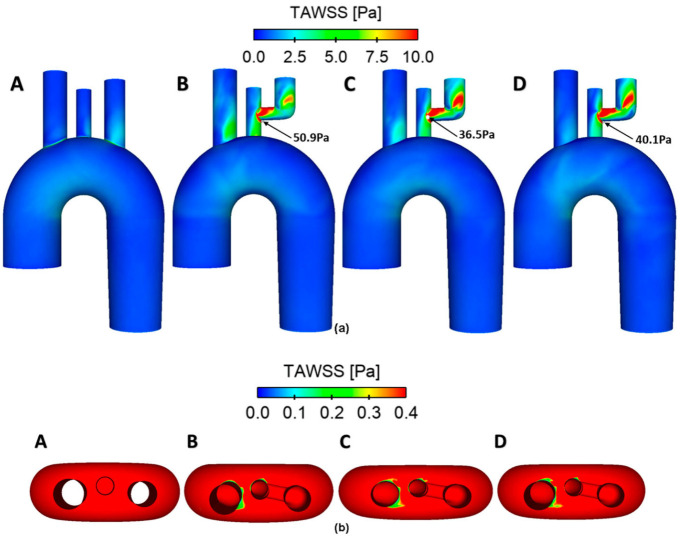
Comparison of TAWSS in the four models: (**A**) non-stented aortic arch (reference case), (**B**) stent-graft 1, (**C**) stent-graft 2, and (**D**) stent-graft 3. TAWSS distributions are displayed in different views to identify areas with (**a**) very high (>10 Pa) and (**b**) very low (<0.4 Pa) values. The maximum TAWSS on the walls of all stented geometries are highlighted by the black arrows.

Predicted TAWSS distributions in the models with and without stent-grafts are shown in Figure 6. It can be seen that the inclusion of the branched stent-graft greatly increased the nonuniformity and magnitude of TAWSS in the arch vessels. The high velocity flow through the inner tunnels impacted on the branch vessel walls when changing its direction, resulting in very high WSS (>10 Pa). In all three stent-graft models, the maximum TAWSS (as indicated by the black arrows) was found at the anastomosis between the bypass graft and the LCCA, with stent-graft 1 showing the highest value of 50.9 Pa. This may cause concerns about the long-term durability at this site since high WSS values are related to graft material fatigue and late device failure [70]. Increasing the inner tunnel diameter clearly reduced the maximum TAWSS values, with ~28% reduction in stent-graft 2 when compared to stent-graft 1. This is because stent-graft 2 has an inner tunnel diameter that closely matches the LCCA diameter, allowing a smooth transition of flow from the inner tunnel to the ICCA. On the other hand, extremely low TAWSS (<0.4 Pa) was observed in regions around the entrance to the supra-aortic vessels of all three stent-graft models (Figure 6b), indicating the potential risk of thrombus formation in these areas because TAWSS values of <0.4 Pa have been suggested to be thrombogenic [58]. Using this idealized model, it was possible to isolate the effect of inner tunnel diameters without the influence of other confounding factors. In the future, a more realistic aortic arch model accounting for its nonplanar curvature can be adopted for similar studies that focus on examining the effect of stent-graft geometric variations.

### 3.2. Patient-Specific Modelling

There have been several patient-specific studies of arch repair, all with varying degrees of limitations and assumptions, availability of data, and different numerical setups. Table 3 shows a summary of these studies and their key findings.

**Figure 7:**
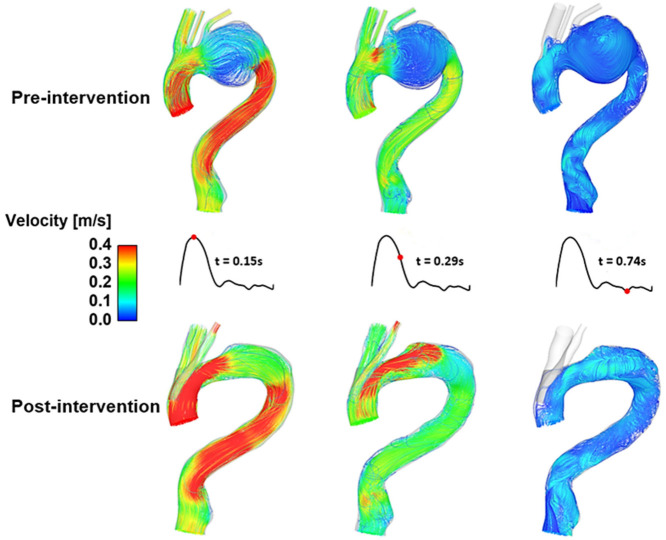
A comparison of instantaneous velocity streamlines in pre- and post-intervention models for TEVAR using a double-branched endograft [72].

An example of our previous work [72] is described here to demonstrate how the aforementioned computational workflow can be applied to patient-specific geometry. Both pre-intervention and post-intervention models were reconstructed from a patient’s CT scans and the numerical simulation results (Figure 7) clearly show changes in flow patterns in the arch region where a large aneurysm was located before intervention. Following TEVAR with a double-branched stent-graft, flow in the arch was dramatically improved with higher velocities and elimination of significant flow recirculation.

**Figure 8:**
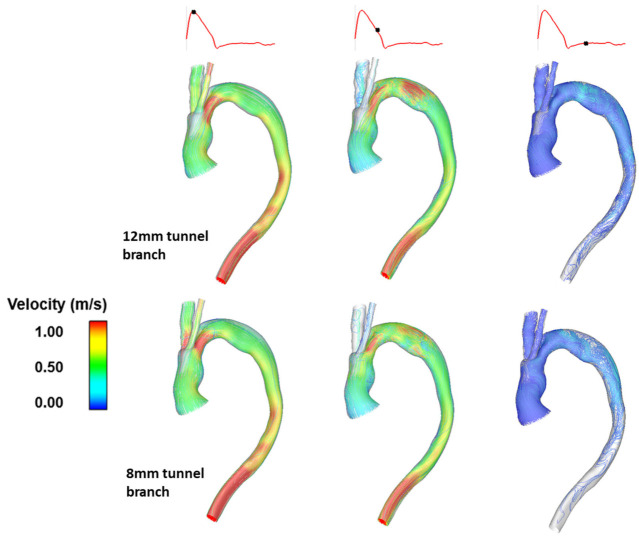
A comparison of instantaneous velocity streamlines in post-TEVAR models for a single patient with different tunnel branch diameters (originally 12 mm, modified to be 8 mm), depicted at peak systole, mid-systolic deceleration and diastole [74].

An example of combining patient-specific geometry with elements of hypothetical modelling is illustrated in Figure 8. The simulated scenarios corresponded to a single patient treated with a double-branched endograft including tunnel branches leading into the emerging arch branches. The original case consisted of tunnel branches of 12 mm in diameter; thereupon, the tunnel branches were artificially altered to be 10 mm and 8 mm in diameter whilst maintaining the features of the overall geometry. This allowed to examine the effect of tunnel branch diameters on flow in the region of interest, whilst keeping the patient-specific characteristics of the overall model.

Predicted flow patterns for the original model with 12 mm tunnel branches and the modified model with 8 mm tunnel branches are compared in Figure 8 where the influence of tunnel branch diameters is clearly demonstrated. In the original model with 12 mm tunnel branches, there was a smoother transition of flow to the IA, without any local acceleration or deceleration in flow velocity. Similarly, the 8 mm tunnel branches in the modified model smoothly guided the flow to the LCCA branch, because they had a similar diameter. This finding is consistent with the simulation results obtained using the idealised model described above, i.e., matching the diameters of the tunnel branch and its respective supra-aortic branch tends to minimize local flow disturbance and produce normal flow transition. Therefore, the diameter of tunnel branches should be selected carefully based on the patient’s arch vessel anatomy.

### 3.3. Future Directions

#### 3.3.1. Virtual Stent-Graft Deployment

**Figure 9:**
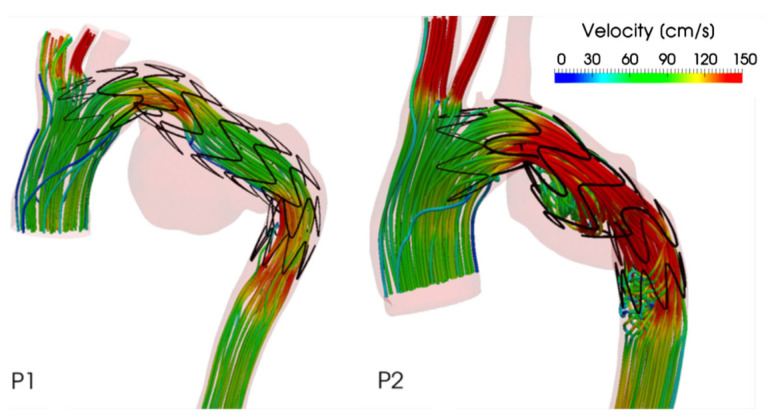
Predicted velocity streamlines with stent-grafts being virtually deployed into two arch aneurysms. Figure was adopted from [85], Copyright (2022), with permission from Elsevier.

One common limitation of all the aforementioned patient-specific CFD studies (Section 3.2) is that the stent-graft walls were assumed to be smooth without considering the presence of stent wires on the stent-graft surface. Stent wires sewn on the endograft surface are exposed to blood flow before intimal growth takes place, which would increase local flow disturbance around the exposed struts during the early days following TEVAR. In most cases, stent wires cannot be segmented from medical images due to the blooming artefacts. A promising approach is to create parametric models of stent-grafts and deploy them into patient-specific aortas using advanced finite element analysis (FEA). This procedure is known as ‘’virtual stent-graft deployment’’ and has recently been applied to the studies of abdominal aortic aneurysms [79,80], type B aortic dissections [81,82] and stenotic coronary arteries [83]. Considering the complex anatomy of the aortic arch, virtual stent-graft deployment in this region would be extremely challenging. Nevertheless, two most recent studies reported virtual simulations of stent-graft deployment in aortic arch aneurysms, with the side branches [84] or without [85]. The FEA methods used in these studies can simulate the actual stenting procedure, including compression, bending and release of the stent-graft so that realistic post-intervention deformed stent-graft configurations can be obtained, which can then be used to perform CFD analysis. Using FEA methods along with CFD simulations will not only allow the post-intervention haemodynamic performance of stent-grafts to be analysed, but also the dynamic interaction between the stent-graft and aortic wall, as well as the effects of various stent designs. As shown in Figure 9, Romarowski et al. applied this hybrid FEA-CFD approach to predict post-intervention haemodynamics in two aortic arch aneurysms [85].

#### 3.3.2. Prediction of Stent-Graft Induced Thrombosis

Thrombosis within the stent-graft may result in occlusion of blood flow to vital side vessels, which could lead to paraplegia or stroke [86]. Thrombus formation and growth was not modelled in the aforementioned simulations of TEAVR for thoracic aortic aneurysms, but WSS related indices were used to identify regions at potential risks of thrombosis. It would be desirable to be able to directly predict stent-graft induced thrombus formation and growth to better evaluate its long-term patency. A shear-driven thrombosis model developed by Menichini and Xu (2016) can be readily incorporated into the haemodynamics models described here [87]. Their model has been tested in patient-specific type B dissection geometries and has shown satisfactory agreement with in vivo observations [88]. However, careful calibration and validation are needed to ensure its suitability for these new applications.

#### 3.3.3. Application to a Large Cohort

All of the aforementioned patient-specific computational studies (Section 3.2) were limited by the small sample size. The branched devices for aortic arch repair are relatively novel designs, which led to limited availability of patient-specific data. Haemodynamic performance within the arch and its branches can be influenced by various factors, such as length, angles between the branches and the main endoprosthesis, as well as length and dimensions of tunnel branches. Therefore, future studies of more patient cases will be needed to examine a wide variety of anatomical scenarios, though some of these have been assessed using either physiologically relevant idealised models [15,67,68], or different stent-graft designs in patient-specific models [39,71,73]. Considering the significant time requirement of computational modelling, future applications of computational flow analyses in a large patient cohort will require automation and optimisation of the workflow and computational procedures. To this end, efforts should be made by taking advantage of artificial intelligence and machine learning techniques [89].

### 3.4. Clinical Relevance

Endografts used in the treatment of aortic arch diseases are at high risk of future complications, such as device migration, endoleak, and subsequent structural failure. All these complications are associated with abnormal flow behaviour and aggressive haemodynamic forces, which can be determined using CFD based on standard clinical images. The aforementioned CFD studies have shown the potential of computational flow modelling in predicting the risk of post-intervention complications. To move this forward, CFD should be used in conjunction with solid mechanics modelling in order to provide a truly comprehensive simulation of physiological conditions. When combined with virtual stent-graft deployment methods (as described in Section 3.3.1), computational modelling can assist clinicians in pre-surgical planning, allowing different stent-graft devices and sizes to be ‘tested’ and evaluated in a patient-specific setting prior to the actual procedure.

## 4. Summary

TEVAR has emerged as a minimally invasive and efficient means of treating aortic arch pathologies. The selection of endografts (in terms of design and size) is a key factor influencing the durability of the device and patient outcomes. Since the goal of TEVAR procedure is to restore normal aortic flow and blood perfusion, and to protect the affected aortic segment against rupture, understanding post-intervention aortic flow patterns and haemodynamic characteristics is of particular interest when evaluating the feasibility of a particular device or procedure. However, not all parameters can be measured in vivo, which calls for the use of in silico computational methods.

Computational modelling has been explored as a tool in determining the haemodynamic performance of endografts and related effects on the vascular system. With the help of CFD to solve equations of conservation of mass and momentum within complex geometrical structures, it is possible to obtain spatially and temporally well-resolved solutions under physiologically realistic conditions. The resolved velocities and pressures can also be used to predict potential complications. The overarching goal of computational modelling in clinical applications is to provide useful information to clinicians to guide them in their treatment planning and surgical interventions, providing the best possible care for patients undergoing the treatment. The use of CFD in conjunction with structural mechanics modelling, can provide a comprehensive range of parameters that may aid in personalized surgical planning and risk stratification for TEVAR.

## Figures and Tables

**Table 2:** Common haemodynamic indices used for computational analyses of aortic arch repair.

Metric	Mathematical Expression	Description
Displacement force [55]	$\;F_{d,i}=\int_{A,i}pdA+\int_{A,i}\left(-\eta_{w}\frac{\partial u_{t}}{\partial n}\right)dA$	Time dependent displacement force due to pressure and friction exerted by the flow of blood on the walls.
Endothelial cell activation potential (ECAP) [56]	$ECAP=\frac{OSI}{TAWSS}$	Synthetic metric to identify regions at a higher risk of thrombus formation.
Helical flow index (HFI) [57]	$HFI=\frac{1}{N_{p}}\overset{N_{p}}{\underset{k=1}{\sum }}\frac{1}{N_{k}}\overset{N_{k}}{\underset{j=1}{\sum }}{\left|LNH\right|}_{j}$	Synthetic descriptor to quantify helicity of particles flowing through the fluid domain.
Oscillatory shear index (OSI) [58]	$OSI=\frac{1}{2}\left(1-\frac{\left|\int_{0}^{T}\tau_{w}dt\right|}{\int_{0}^{T}\left|\tau_{w}\;\right|dt}\right)$	Change of direction of the wall shear stress (WSS) vector from the primary direction of flow.
Platelet activation potential (PLAP) [40]	$PLAP=\int_{t-2T}^{t}\left|D\left(x\left(\tau \right),\tau \right)\right|d\tau$	Non-dimensional scalar index of magnitudes of shear rate that particles accumulate travelling through the domain.
Time-averaged WSS (TAWSS) [58]	$TAWSS=\frac{1}{T}\int_{0}^{T}\left|\tau_{w}\;\right|dt$	Average of the WSS magnitude over the cardiac cycle.
Relative residence time (RRT) [58]	$RRT=\frac{1}{\left(1-2*OSI\right)*TAWSS}$	Amount of time that solutes and particles of the blood may spend near the vessel wall.
Transverse WSS [59]	$TransWSS=\frac{1}{T}\int_{0}^{T}\left|\tau_{w}\;.\;\left(n\;x\;\frac{\int_{0}^{T}\tau_{w}dt}{\left|\int_{0}^{T}\tau_{w}dt\right|}\;\right)\right|dt$	Average over the cardiac cycle of WSS components perpendicular to the temporal mean WSS vector.
Turbulence intensity (*Tu*) [60]	$Tu=\frac{\sqrt{\frac{2}{3}k}}{V}$	Used to measure the level of turbulence and disturbance in flow.
Vorticity [61]	ω=∇×v	Vector field that describes the circulation per unit area at a point in a fluid flow field.
*T* is the time period of a cardiac cycle; *τ_w_* is the wall shear stress vector; *v_x_*, *v_y_*, *v_z_* are the velocity fields in the *x*, *y*, and *z* components; *k* is the turbulence kinetic energy; *V* is the instantaneous localised velocity, LNH is the local normalised helicity, $\left|D\left(x\left(\tau \right),\tau \right)\right|$ is the Frobenius norm of the symmetric part of the spatial gradient of the velocity tensor.		

**Table 3:** A summary of computational studies carried out using patient-specific aortic geometries.

Authors	Year	Landing Zone	Condition and Treatment/Device	Key Findings
Midulla et al. [61]	2021	0–4	▪ Twenty patients with various thoracic aortic diseases. ▪ TEVAR with endografts being landed in Z0 – 4.	▪ Increased WSS and vorticity in some cases post-procedure. ▪ WSS median values increased from 4.19 Pa to 4.90 Pa and vorticity median values decreased from 40.38 Hz to 39.17 Hz.
van Bakel et al. [39]	2018	0	▪ Patient with a saccular aortic arch aneurysm. ▪ 4 different branched endografts designs.	▪ 2 antegrade inner branches performed favourable with higher blood flow and lower blood shear rate in the cervical arteries. ▪ Single retrograde inner branch was least favourable.
Chiu et al. [71]	2018	0	▪ Patient with an aortic arch aneurysm. ▪ 5 branched endografts with different inner side branches’ configurations.	▪ Flow distributions to the arch vessels were comparable between all simulated models except for one with an antegrade LSCA side branch. ▪ Internal side branches caused more oscillatory and helical blood flow, which may further result in blood clot formation.
Zhu et al. [72]	2019	0	▪ Two patients with aortic arch aneurysms. ▪ Treated with double-branched thoracic endograft.	▪ Sufficient blood perfusion with increased WSS in the aortic arch, which may help reduce the risk of thrombus formation. ▪ Increased WSS asymmetry and flow derangement in the ascending aorta due to the inner tunnels may have a detrimental effect in the long-term. ▪ Maximum flow-induced displacement forces on the endografts were well below the threshold for device migration.
Xiong et al. [73]	2020	0	▪ Non-A-non-B aortic dissection. ▪ Type I hybrid arch repair along with a subsequent TEVAR.	▪ Regular blood flow in the TL but still disturbed in the FL. ▪ Usual blood supply to most of the aortic branches observed and luminal pressure difference reduced by 58.2% at 1-year follow-up. ▪ Regions with abnormal TAWSS significantly decreased in the TL but remained high in the FL.
Sengupta et al. [74]	2022	0	▪ Two patients with TAAD. ▪ Treated with TEVAR in Z0 using a double-branched endograft for TAAD were included.	▪ Normal flow patterns observed in both patients following the TEVAR procedure with variations in inner tunnel branch diameters having noticeable influence on local flow patterns and WSS oscillations. ▪ Tunnel branch diameters should be as close to the respective arch vessels as possible to avoid any adverse clinical consequences.
Qiao et al. [75]	2019	1	▪ Two patients with TBAD and an aortic arch aneurysm, respectively were included ▪ Treated with TEVAR in Z1, whereas the blood perfusion to the LCCA and LSCA were rebuilt by the in situ fenestration (ISF) technique.	▪ Wall pressures of the LCCA and LSCA found to be relatively low. ▪ Pressure difference between the inner and outer surfaces of the protruding segment of the LSCA fenestration might lead to stent migration. ▪ Regions with low TAWSS but high OSI and RRT were observed at the aortic arch distal to the LSCA fenestration making it prone to thrombus formation.
Auricchio et al. [76]	2014	2	▪ TBAD patient. ▪ Treated with TEVAR involving LSCA occlusion.	▪ Bird-beak configuration in the proximal end of endograft caused significant flow disturbance and pressure drop ▪ Similar results were also observed in the distal thoracic aorta as a result of lumen narrowing.
Nauta et al. [40]	2017	2	▪ Patient presenting with the distal aortic arch aneurysm. ▪ Treated with TEVAR in Z2 where the root of LSCA was occluded.	▪ High PLAP values were found at the thrombus location of the post-TEVAR model. ▪ PLAP was reduced by 37% and 20% through a surgical repair of the ascending aorta and a single-branched endograft restoring blood perfusion into the LSCA, respectively. ▪ Virtual repair strategy using a conformable endograft had limited effect on the predicted PLAP.
Van Bakel et al. [77]	2018	2	▪ Three patients with thoracic aortic aneurysm and one patient with penetrating aortic ulcer. ▪ Treated with TEVAR in landing Z2.	▪ Mean flow in the LCCA increased significantly to account for the blood supply to the LSCA via a separate bypass procedure. ▪ Maximum flow velocity in the LCCA increased by 62%. ▪ Displacement forces experienced by the endografts were positively correlated to their surface areas, and high displacement forces in one patient might cause Type I endoleaks at one-year follow up.
Tricarico et al. [78]	2020	2	▪ Patient presenting with TAA. ▪ Treated with and LSCA-branched endograft.	▪ Longer intra-aortic protrusion lengths of the LSCA branch resulted in larger pressure drops and energy losses across the stented LSCA, as well as greater areas of peak WSS along the outer surface of the LSCA internal branch. ▪ It associated with higher risk of thrombus formation due to the more disturbed blood flow, while a protrusion length of less than 5 mm could reduce the risk of thrombosis.
FL—False lumen, OSI—Oscillatory shear index, PLAP—Platelet activation potential, RRT—Relative residence time, TAWSS—Time-averaged wall shear stress, TL—True lumen, WSS—Wall shear stress, TBAD—Type B aortic dissection, TEVAR—Thoracic endovascular aortic repair				

## Data Availability

The data presented in this study are available on request from the corresponding author.

## References

[1] Desai N.D., Roselli E.E. (2018). Complex aortic arch surgery. Ann. Cardiothorac. Surg..

[2] Cherrie Z.A., Victor M.R. (2015). Upcoming Technology for Aortic Arch Aneurysms. Endovasc. Today.

[3] Stankovic Z., Allen B.D., Garcia J., Jarvis K.B., Markl M. (2014). 4D flow imaging with MRI. Cardiovasc. Diagn. Ther..

[4] Takahashi K., Sekine T., Ando T., Ishii Y., Kumita S. (2021). Utility of 4D flow MRI in thoracic aortic diseases: A Literature Review of Clinical Applications and Current Evidence. Magn. Reson. Med. Sci..

[5] Cheng Z., Juli C., Wood N.B., Gibbs R.G.J., Xu X.Y. (2014). Predicting flow in aortic dissection: Comparison of computational model with PC-MRI velocity measurements. Med. Eng. Phys..

[6] Armour C.H., Guo B., Saitta S., Pirola S., Liu Y., Dong Z., Xu X.Y. (2022). Evaluation and verification of patient-specific modelling of type B aortic dissection. Comput. Biol. Med..

[7] Pirola S., Guo B., Menichini C., Saitta S., Fu W., Dong Z., Xu X.Y. (2019). 4-D flow MRI-based computational analysis of blood flow in patient-specific aortic dissection. IEEE Trans. Biomed. Eng..

[8] Guyton A.C., Hall J.E. (1986). Textbook of Medical Physiology.

[9] Booher A.M., Eagle K.A. (2011). Diagnosis and management issues in thoracic aortic aneurysm. Am. Heart J..

[10] Clouse W.D., Hallett J.W., Schaff H.V., Spittell P.C., Rowland C.M., Ilstrup D.M., Melton L.J. (2004). Acute Aortic Dissection: Population-Based Incidence Compared With Degenerative Aortic Aneurysm Rupture. Mayo Clin. Proc..

[11] Hebballi R., Swanevelder J. (2009). Diagnosis and management of aortic dissection. Contin. Educ. Anaesth. Crit. Care Pain.

[12] Shirakawa Y., Kuratani T., Shimamura K., Torikai K., Sakamoto T., Shijo T., Sawa Y. (2014). The efficacy and short-term results of hybrid thoracic endovascular repair into the ascending aorta for aortic arch pathologies. Eur. J. Cardio-Thorac. Surg..

[13] Nienaber C.A., Fattori R., Lund G., Dieckmann C., Wolf W., von Kodolitsch Y., Nicolas V., Pierangeli A. (1999). Nonsurgical Reconstruction of Thoracic Aortic Dissection by Stent–Graft Placement. N. Engl. J. Med..

[14] Bodell B.D., Taylor A.C., Patel P.J. (2018). Thoracic Endovascular Aortic Repair: Review of Current Devices and Treatments Options. Tech. Vasc. Interv. Radiol..

[15] Nardi A., Avrahami I. (2017). Approaches for treatment of aortic arch aneurysm, a numerical study. J. Biomech..

[16] Makaroun M.S., Dillavou E.D., Wheatley G.H., Cambria R.P. (2008). Five-year results of endovascular treatment with the Gore TAG device compared with open repair of thoracic aortic aneurysms. J. Vasc. Surg..

[17] Martin G., Riga C., Gibbs R., Jenkins M., Hamady M., Bicknell C. (2016). Short- and Long-term Results of Hybrid Arch and Proximal Descending Thoracic Aortic Repair: A Benchmark for New Technologies. J. Endovasc. Ther..

[18] Naughton P.A., Park M.S., Morasch M.D., Rodriguez H.E., Garcia-Toca M., Wang C.E., Eskandari M.K. (2012). Emergent Repair of Acute Thoracic Aortic Catastrophes: A Comparative Analysis. Arch Surg..

[19] Ishimaru S. (2004). Endografting of the Aortic Arch. J. Endovasc. Ther..

[20] Waterford S.D., Chou D., Bombien R., Uzun I., Shah A., Khoynezhad A. (2016). Left Subclavian Arterial Coverage and Stroke During Thoracic Aortic Endografting: A Systematic Review. Ann. Thorac. Surg..

[21] Zamor K.C., Eskandari M.K., Rodriguez H.E., Ho K.J., Morasch M.D., Hoel A.W. (2015). Outcomes of Thoracic Endovascular Aortic Repair and Subclavian Revascularization Techniques. J. Am. Coll. Surg..

[22] Criado F.J., Clark N.S., Barnatan M.F. (2002). Stent graft repair in the aortic arch and descending thoracic aorta: A 4-year experience. J. Vasc. Surg..

[23] Ueda T., Fleischmann D., Dake M.D., Rubin G.D., Sze D.Y. (2010). Incomplete endograft apposition to the aortic arch: Bird-beak configuration increases risk of endoleak formation after thoracic endovascular aortic repair. Radiology.

[24] Balm R., Reekers J.A., Jacobs M.J. (2000). Classification of endovascular procedures for treating thoracic aortic aneurysms. Surgical and Endovascular Treatment of Aortic Aneurysms.

[25] Antoniou G.A., Sakka K.M.E., Hamady M., Wolfe J.H.N. (2010). Hybrid Treatment of Complex Aortic Arch Disease with Supra-aortic Debranching and Endovascular Stent Graft Repair. Eur. J. Vasc. Endovasc. Surg..

[26] Zerwes S., Leissner G., Gosslau Y., Jakob R., Bruijnen H.-K., Oertl F., Woelfle K. (2015). Clinical outcomes in hybrid repair procedures for pathologies involving the aortic arch. Vascular.

[27] Iba Y., Minatoya K., Matsuda H., Sasaki H., Tanaka H., Oda T., Kobayashi J. (2014). How should aortic arch aneurysms be treated in the endovascular aortic repair era? A risk-adjusted comparison between open and hybrid arch repair using propensity score-matching analysis. Eur. J. Cardio-Thorac. Surg..

[28] Benedetto U., Melina G., Angeloni E., Codispoti M., Sinatra R. (2013). Current results of open total arch replacement versus hybrid thoracic endovascular aortic repair for aortic arch aneurysm: A meta-analysis of comparative studies. J. Thorac. Cardiovasc. Surg..

[29] Tokuda Y., Oshima H., Narita Y., Abe T., Araki Y., Mutsuga M., Fujimoto K., Terazawa S., Yagami K., Ito H. (2016). Hybrid versus open repair of aortic arch aneurysms: Comparison of postoperative and mid-term outcomes with a propensity score-matching analysis. Eur. J. Cardio-Thorac. Surg..

[30] Heaton D.H. (2009). The Next Generation of Aortic Endografts. Endovasc. Today.

[31] Haulon S., Greenberg R.K., Spear R., Eagleton M., Abraham C., Lioupis C., Verhoeven E., Ivancev K., Kolbel T., Stanley B. (2014). Global experience with an inner branched arch endograft. J. Thorac. Cardiovasc. Surg..

[32] Spear R., Haulon S., Ohki T., Tsilimparis N., Kanaoka Y., Milne C.P.E., Debus S., Takizawa R., Kölbel T. (2016). Editor’s Choice—Subsequent Results for Arch Aneurysm Repair with Inner Branched Endografts. Eur. J. Vasc. Endovasc. Surg..

[33] Czerny M., Rylski B., Morlock J., Schröfel H., Beyersdorf F., Saint Lebes B., Meyrignac O., Mokrane F., Lescan M., Schlensak C. (2018). Orthotopic branched endovascular aortic arch repair in patients who cannot undergo classical surgery. Eur. J. Cardio Thorac. Surg..

[34] van Bakel T.M., de Beaufort H.W., Trimarchi S., Marrocco-Trischitta M.M., Bismuth J., Moll F.L., Patel H.J., van Herwaarden J.A. (2018). Status of branched endovascular aortic arch repair. Ann. Cardiothorac. Surg..

[35] Ferrer C., Cao P. (2018). Endovascular arch replacement with a dual branched endoprosthesis. Ann. Cardiothorac. Surg..

[36] Ong C., Xiong F., Kabinejadian F., Praveen Kumar G., Cui F., Chen G., Ho P., Leo H. (2019). Hemodynamic analysis of a novel stent graft design with slit perforations in thoracic aortic aneurysm. J. Biomech..

[37] Tazaki J., Inoue K., Higami H., Higashitani N., Toma M., Saito N., Kawatou M., Kimura T. (2017). Thoracic endovascular aortic repair with branched Inoue Stent Graft for arch aortic aneurysms. J. Vasc. Surg..

[38] Czerny M., Berger T., Kondov S., Siepe M., Saint Lebes B., Mokrane F., Rousseau H., Lescan M., Schlensak C., Andic M. (2021). Results of endovascular aortic arch repair using the Relay Branch system. Eur. J. Cardio Thorac. Surg..

[39] van Bakel T.M., Arthurs C.J., van Herwaarden J.A., Moll F.L., Eagle K.A., Patel H.J., Trimarchi S., Figueroa C.A. (2018). A computational analysis of different endograft designs for Zone 0 aortic arch repair. Eur. J. Cardio-Thorac. Surg..

[40] Nauta F.J., Lau K.D., Arthurs C.J., Eagle K.A., Williams D.M., Trimarchi S., Patel H.J., Figueroa C.A. (2017). Computational fluid dynamics and aortic thrombus formation following thoracic endovascular aortic repair. Ann. Thorac. Surg..

[41] Santos I.C., Rodrigues A., Figueiredo L., Rocha L.A., Tavares J.M.R. (2012). Mechanical properties of stent–graft materials. Proc. Inst. Mech. Eng..

[42] Pirola S., Cheng Z., Jarral O.A., O’Regan D.P., Pepper J.R., Athanasiou T., Xu X.Y. (2017). On the choice of outlet boundary conditions for patient-specific analysis of aortic flow using computational fluid dynamics. J. Biomech..

[43] Buchanan J.R., Kleinstreuer C., Comer J.K. (2000). Rheological effects on pulsatile hemodynamics in a stenosed tube. Comput. Fluids.

[44] Popel A.S., Enden G. (1993). An analytical solution for steady flow of a Quemada fluid in a circular tube. Rheol. Acta.

[45] Biasetti J., Gasser T.C., Auer M., Hedin U., Labruto F. (2010). Hemodynamics of the normal aorta compared to fusiform and saccular abdominal aortic aneurysms with emphasis on a potential thrombus formation mechanism. Ann. Biomed. Eng..

[46] Perktold K., Resch M., Florian H. (1991). Pulsatile Non-Newtonian Flow Characteristics in a Three-Dimensional Human Carotid Bifurcation Model. J. Biomech. Eng..

[47] Cho Y.I., Kensey K.R. (1991). Effects of the Non-Newtonian Viscosity of Blood on Flows in a Diseased Arterial Vessel. Part 1: Steady Flows. Biorheology.

[48] Lee S.-W., Steinman D.A. (2007). On the relative importance of rheology for image-based cfd models of the carotid bifurcation. J. Biomech. Eng..

[49] Nguyen K.T., Clark C.D., Chancellor T.J., Papavassiliou D.V. (2008). Carotid geometry effects on blood flow and on risk for vascular disease. J. Biomech..

[50] Polanczyk A., Podyma M., Stefanczyk L., Szubert W., Zbicinski I. (2015). A 3D model of thrombus formation in a stent-graft after implantation in the abdominal aorta. J. Biomech..

[51] Holmlund P. (2013). Computational Fluid Dynamic Simulations of Pulsatile Flow in Stenotic Vessel Models. Master’s Thesis.

[52] Wood N.B. (1999). Aspects of Fluid Dynamics Applied to the Larger Arteries. J. Theor. Biol..

[53] Kousera C.A., Wood N.B., Seed W.A., Torii R., O’Regan D., Xu X.Y. (2013). A Numerical Study of Aortic Flow Stability and Comparison With In Vivo Flow Measurements. J. Biomech. Eng..

[54] Nerem R.M., Seed W.A., Wood N.B. (1972). An experimental study of the velocity distribution and transition to turbulence in the aorta. J. Fluid Mech..

[55] Kandail H., Hamady M., Xu X.Y. (2014). Patient-Specific Analysis of Displacement Forces Acting on Fenestrated Stent Grafts for Endovascular Aneurysm Repair. J. Biomech..

[56] Di Achille P., Tellides G., Figueroa C.A., Humphrey J.D. (2014). A Haemodynamic Predictor of Intraluminal Thrombus Formation in Abdominal Aortic Aneurysms. Proc. Math. Phys. Eng..

[57] Morbiducci U., Ponzini R., Rizzo G., Cadioli M., Esposito A., De Cobelli F., Del Maschio A., Montevecchi F.M., Redaelli A. (2009). In Vivo Quantification of Helical Blood Flow in Human Aorta by Time-Resolved Three-Dimensional Cine Phase Contrast Magnetic Resonance Imaging. Ann. Biomed. Eng..

[58] Suess T., Anderson J., Danielson L., Pohlson K., Remund T., Blears E., Gent S., Kelly P. (2016). Examination of Near-Wall Hemodynamic Parameters in the Renal Bridging Stent of Various Stent Graft Configurations for Repairing Visceral Branched Aortic Aneurysms. J. Vasc. Surg..

[59] Mohamied Y., Sherwin S.J., Weinberg P.D. (2017). Understanding the fluid mechanics behind transverse wall shear stress. J. Biomech..

[60] Tan F.P.P., Borghi A., Mohiaddin R.H., Wood N.B., Thom S., Xu X.Y. (2009). Analysis of flow patterns in a patient-specific thoracic aortic aneurysm model. Comput. Struct..

[61] Midulla M., Moreno R., Negre-Salvayre A., Beregi J.-P., Haulon S., Loffroy R., Dake M., Rousseau H. (2021). Impact of Thoracic Endografting on the Hemodynamics of the Native Aorta: Pre-and Postoperative Assessments of Wall Shear Stress and Vorticity Using Computational Fluid Dynamics. J. Endovasc. Ther..

[62] Malek A.M., Alper S.L., Izumo S. (1999). Hemodynamic Shear Stress and Its Role in Atherosclerosis. JAMA.

[63] Nobili M., Sheriff J., Morbiducci U., Redaelli A., Bluestein D. (2008). Platelet Activation Due to Hemodynamic Shear Stresses: Damage Accumulation Model and Comparison to in Vitro Measurements. ASAIO J..

[64] Ekaterinaris J.A., Ioannou C.V., Katsamouris A.N. (2006). Flow Dynamics in Expansions Characterizing Abdominal Aorta Aneurysms. Ann. Vasc. Surg..

[65] Fry D.L. (1969). Certain Histological and Chemical Responses of the Vascular Interface to Acutely Induced Mechanical Stress in the Aorta of the Dog. Circ. Res..

[66] Liu Z., Teng S., Chen G., Wu L., Yang J., Cui F., Ho P. (2021). A systematic approach to further improve stent-graft performance. Mater. Des..

[67] Liu J., Cai X., Zhan Y., Zhu H., Ao H., Wan Y., Luo H., Yang Z., Zhang Q. (2022). Hemodynamic evaluation of different stent graft schemes in aortic arch covered stent implantation. Med. Nov. Technol. Devices.

[68] Zhu Y. (2020). Computational Analysis of the Hemodynamic Performance of Novel Endovascular and Surgical Procedures for Complex Aortic Diseases. Ph.D. Thesis.

[69] Finlay A., Johnson M., Forbes T.L. (2012). Surgically Relevant Aortic Arch Mapping Using Computed Tomography. Ann. Vasc. Surg..

[70] Chatzizisis Y.S., Coskun A.U., Jonas M., Edelman E.R., Feldman C.L., Stone P.H. (2007). Role of Endothelial Shear Stress in the Natural History of Coronary Atherosclerosis and Vascular Remodeling: Molecular, Cellular, and Vascular Behavior. J. Am. Coll. Cardiol..

[71] Chiu T.L., Tang A.Y.S., Cheng S.W.K., Chow K.W. (2018). Analysis of flow patterns on branched endografts for aortic arch aneurysms. Inform. Med. Unlocked.

[72] Zhu Y., Zhan W., Hamady M., Xu X.Y. (2019). A pilot study of aortic hemodynamics before and after thoracic endovascular repair with a double-branched endograft. Med. Nov. Technol. Devices.

[73] Xiong Z., Yang P., Li D., Qiu Y., Zheng T., Hu J. (2020). A computational fluid dynamics analysis of a patient with acute non-A-non-B aortic dissection after type I hybrid arch repair. Med. Eng. Phys..

[74] Sengupta S., Hamady M., Xu X.-Y. (2022). Haemodynamic Analysis of Branched Endografts for Complex Aortic Arch Repair. Bioengineering.

[75] Qiao Y., Mao L., Ding Y., Fan J., Zhu T., Luo K. (2020). Hemodynamic consequences of TEVAR with in situ double fenestrations of left carotid artery and left subclavian artery. Med. Eng. Phys..

[76] Auricchio F., Conti M., Lefieux A., Morganti S., Reali A., Sardanelli F., Secchi F., Trimarchi S., Veneziani A. (2014). Patient-specific analysis of post-operative aortic hemodynamics: A focus on thoracic endovascular repair (TEVAR). Comput. Mech..

[77] van Bakel T.M., Romarowski R.M., Morganti S., van Herwaarden J.A., Moll F.L., de Beaufort H.W., Marrocco-Trischitta M.M., Secchi F., Conti M., Auricchio F. (2018). Blood flow after endovascular repair in the aortic arch: A computational analysis. Aorta.

[78] Tricarico R., Tran-Son-Tay R., Laquian L., Scali S.T., Lee T.-C., Beck A.W., Berceli S.A., He Y. (2020). Haemodynamics of different configurations of a left subclavian artery stent graft for thoracic endovascular aortic repair. Eur. J. Vasc. Endovasc. Surg..

[79] De Bock S., Iannaccone F., De Santis G., De Beule M., Van Loo D., Devos D., Vermassen F., Segers P., Verhegghe B. (2012). Virtual Evaluation of Stent Graft Deployment: A Validated Modeling and Simulation Study. J. Mech. Behav. Biomed. Mater..

[80] Perrin D., Badel P., Orgéas L., Geindreau C., Dumenil A., Albertini J.-N., Avril S. (2015). Patient-Specific Numerical Simulation of Stent-Graft Deployment: Validation on Three Clinical Cases. J. Biomech..

[81] Chen D., Wei J., Deng Y., Xu H., Li Z., Meng H., Han X., Wang Y., Wan J., Yan T. (2018). Virtual Stenting with Simplex Mesh and Mechanical Contact Analysis for Real-Time Planning of Thoracic Endovascular Aortic Repair. Theranostics.

[82] Kan X., Ma T., Lin J., Wang L., Dong Z., Xu X.Y. (2021). Patient-specific simulation of stent-graft deployment in type B aortic dissection: Model development and validation. Biomech. Model. Mechanobiol..

[83] Wei L., Leo H.L., Chen Q., Li Z. (2019). Structural and Hemodynamic Analyses of Different Stent Structures in Curved and Stenotic Coronary Artery. Front. Bioeng. Biotechnol..

[84] Derycke L., Perrin D., Cochennec F., Albertini J.-N., Avril S. (2019). Predictive numerical simulations of double branch stent-graft deployment in an aortic arch aneurysm. Ann. Biomed. Eng..

[85] Romarowski R.M., Faggiano E., Conti M., Reali A., Morganti S., Auricchio F. (2019). A novel computational framework to predict patient-specific hemodynamics after TEVAR: Integration of structural and fluid-dynamics analysis by image elaboration. Comput. Fluids.

[86] Buth J., Harris P.L., Hobo R., van Eps R., Cuypers P., Duijm L., Tielbeek X. (2007). Neurologic Complications Associated with Endovascular Repair of Thoracic Aortic Pathology: Incidence and Risk Factors. A Study from the European Collaborators on Stent/Graft Techniques for Aortic Aneurysm Repair (EUROSTAR) Registry. J. Vasc. Surg..

[87] Menichini C., Xu X.Y. (2016). Mathematical modeling of thrombus formation in idealized models of aortic dissection: Initial findings and potential applications. J. Math. Biol..

[88] Menichini C., Cheng Z., Gibbs R.G.J., Xu X.Y. (2018). A Computational Model for False Lumen Thrombosis in Type B Aortic Dissection Following Thoracic Endovascular Repair. J. Biomech..

[89] Arzani A., Wang J.-X., Sacks M.S., Shadden S.C. (2022). Machine Learning for Cardiovascular Biomechanics Modeling: Challenges and Beyond. Ann. Biomed. Eng..

